# Pleiotropic fitness effects of a *Drosophila* odorant-binding protein

**DOI:** 10.1093/g3journal/jkac307

**Published:** 2022-12-01

**Authors:** Sneha S Mokashi, Vijay Shankar, Joel A Johnstun, Trudy F C Mackay, Robert R H Anholt

**Affiliations:** Department of Genetics and Biochemistry and Center for Human Genetics, Clemson University, 114 Gregor Mendel Circle, Greenwood, SC 29646, USA; Department of Genetics and Biochemistry and Center for Human Genetics, Clemson University, 114 Gregor Mendel Circle, Greenwood, SC 29646, USA; Department of Biological Sciences, Program in Genetics, North Carolina State University, Raleigh, NC 27695, USA; Department of Genetics and Biochemistry and Center for Human Genetics, Clemson University, 114 Gregor Mendel Circle, Greenwood, SC 29646, USA; Department of Genetics and Biochemistry and Center for Human Genetics, Clemson University, 114 Gregor Mendel Circle, Greenwood, SC 29646, USA

**Keywords:** *Obp56h*, CRISPR-Cas9, pleiotropy, fitness, starvation resistance, heat stress, RNA sequencing, chemosensation, sleep, sexual dimorphism

## Abstract

Insect odorant-binding proteins (OBPs) are members of a rapidly evolving multigene family traditionally thought to facilitate chemosensation. However, studies on *Drosophila* have shown that members of this family have evolved functions beyond chemosensation, as evident from their expression in reproductive tissues and the brain. Previous studies implicated diverse functions of *Obp56h*, a member of the largest gene cluster of the *D. melanogaster Obp* repertoire. Here, we examined the effect of CRISPR/*Cas9*-mediated deletion of *Obp56h* on 2 fitness phenotypes, on resistance to starvation stress and heat stress, and on locomotion and sleep phenotypes. *Obp56h*^−/−^ mutants show a strong sexually dimorphic effect on starvation stress survival, with females being more resistant to starvation stress than the control. In contrast, *Obp56h*^−/−^ females, but not males, are highly sensitive to heat stress. Both sexes show changes in locomotion and sleep patterns. Transcriptional profiling of RNA from heads of *Obp56h*^−/−^ flies and the wildtype control reveals differentially expressed genes, including gene products associated with antimicrobial immune responses and members of the *Turandot* family of stress-induced secreted peptides. In addition, differentially expressed genes of unknown function were identified in both sexes. Genes encoding components of the mitochondrial electron transport chain, cuticular proteins, gene products associated with regulation of feeding behavior (*Lst* and *CCHa2*), ribosomal proteins, lncRNAs, snoRNAs, tRNAs, and snRNAs show changes in transcript abundances in *Obp56h*^−/−^ females. These differentially expressed genes are likely to contribute to *Obp56h*-mediated effects on the diverse phenotypes that arise upon deletion of this OBP.

## Introduction

Multigene families that arise through repeated gene duplication events are one of the key drivers of adaptive evolution (Long *et al*., 2013; Magadum *et al*., 2013; Vaschetto and Ortiz, 2019). Functional redundancy within such gene families confers stability in the face of environmental fluctuations (Zhou *et al*., 2012), and relaxed selection pressures on daughter genes allow neofunctionalization and functional diversification. It is generally accepted that the diverse family of insect odorant-binding proteins (OBPs) evolved rapidly to facilitate chemosensation by promoting solubilization of airborne odorants in the perilymph surrounding olfactory sensory neurons (Hekmat-Scafe *et al*., 2002; Vieira *et al*., 2007; Pelosi *et al*., 2014; Sun, Xiao, *et al.*, 2018). Although CRISPR-mediated deletion of a subset of *Drosophila melanogaster Obp* genes did not affect electrophysiological responses to odorants (Xiao *et al*., 2019), behavioral measurements using RNAi knockdown of *Obp*s implicated combinatorial interactions between odorants and OBPs (Swarup *et al*., 2011), underscoring functional redundancy among members of this multigene family.

In addition to their role in olfaction, studies in *D. melanogaster* have shown that members of the *Obp* gene family have been co-opted for functions other than chemosensation. *Obp59a* has been implicated in humidity sensing (Sun, Larter, *et al.*, 2018). CRISPR-mediated deletion of the *Obp50a-d* gene cluster followed by reinsertion of different combinations of paralogs provided evidence for functional diversification, redundancy, and epistasis among paralogs of this cluster and implicated *Obp50a* in development and *Obp50d* in stress resistance (Johnstun *et al*., 2021). Association analyses with wild-derived inbred *D. melanogaster* lines of the *D. melanogaster* genetic reference panel identified two polymorphic markers in *Obp19d* that were associated with variation in lifespan (Arya *et al*., 2010). A paralog of *Obp19d*, *Obp19c*, is expressed in ovaries and has been implicated in oviposition and postmating behavior (Arya *et al*., 2010). In addition, *Obp8a* is abundant in the male accessory gland. These observations suggest that OBP8a and OBP19c, and potentially other OBPs found in seminal fluid (McGraw *et al*., 2004; Findlay *et al*., 2008), may bind thus far unidentified hydrophobic molecules associated with the transfer of sperm during mating and stimulation of oviposition.

The *Obp56* cluster is the largest gene cluster of the multigene *Obp* family in *D. melanogaster*. Within this cluster, *Obp56h* provides an ideal target for CRISPR/*Cas9* gene editing, since it is a small gene (651 bp) without nested genes, and the nearest genes are 12,891 bp upstream and 10,374 bp downstream. RNA interference of *Obp56h* affects olfactory behavior (Swarup *et al*., 2011), avoidance of bitter tastants (Swarup *et al*., 2014), mating behavior (Shorter *et al*., 2016), and expression of co-regulated genes associated with lipid metabolism, immune/defense response, and heat stress (Shorter *et al*., 2016). *Obp56h* is expressed in the antenna (Galindo and Smith, 2001) and labellum (Galindo and Smith, 2001) and in cells of the central brain (Baker *et al*., 2021; Mokashi *et al*., 2021), which suggests functional pleiotropy at the *Obp56h* locus, i.e. diverse physiological roles of its gene product. Here, we show that CRISPR/*Cas9*-mediated deletion of *Obp56h* reveals sexually dimorphic effects on starvation stress resistance, resistance to heat stress, locomotion, and sleep patterns. In addition to effects on organismal phenotypes, transcriptional profiling shows differentially expressed genes between *Obp56h*^−/−^ flies and the *Canton S-B* (*CSB*) control, associated with the diverse *Obp56h*-related phenotypes that are affected upon deletion of this odorant binding protein.

## Materials and methods

### Drosophila lines

**Fig. 1.**
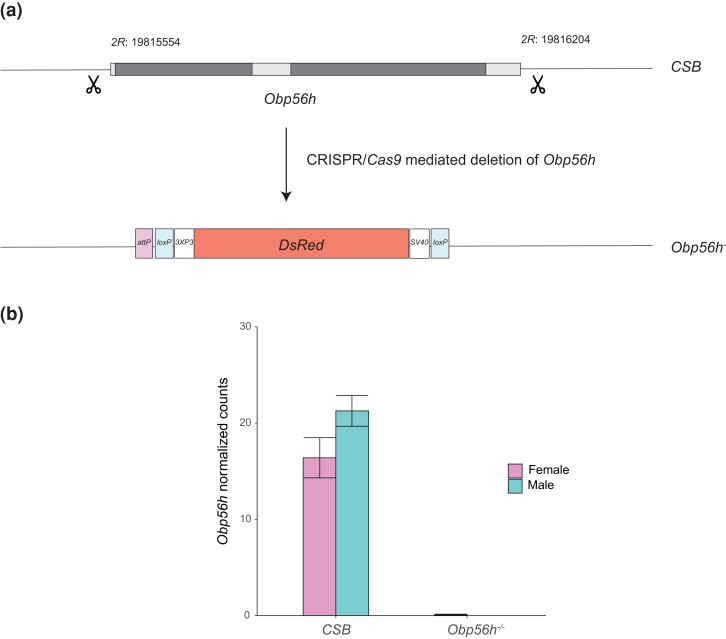
CRISPR/*Cas*9-mediated deletion of *Obp56h*. a) Construction of the *Obp56h* null allele. Dark boxes represent exons of the *Obp56h* gene, and light boxes indicate the intron and 5′ and 3′ untranslated sequences. We designed guide RNAs flanking the *Obp56h* gene for CRISPR/*Cas*9-mediated deletion at the cut sites, indicated by the scissor symbols, in the *CSB* genetic background (Supplementary Table 1). We replaced the gene with a cassette that contains a *DsRed* fluorescent marker under the control of an eyespecific *3XP3* promoter and with SV40 polyadenylation sequences, *loxP* sites for *Cre*-mediated removal of the insert, and an *attP* site. b) Average normalized *Obp56h* cpm expression counts from whole genome RNA sequencing for males and females for *Obp56h* deletion flies and the *CSB* control (see also Supplementary Tables 3 and 4).

To generate a CRISPR/*Cas9*-mediated null allele of *Obp56h* in a *CSB* genetic background, we designed two guide RNAs flanking the gene using the Optimal Target Finder online tool (Gratz *et al*., 2014; Supplementary Table 1; Fig. 1a) and cloned them into the *pU6-Bbs1-chiRNA* plasmid. We used the *pBS-Hsp70-Cas9* plasmid as a source for *Cas9* and generated a donor plasmid containing *3XP3*-driven *DsRed* flanked by 1kb sequences homologous to the regions flanking the *Obp56h* gene. This vector also has *loxP* sites flanking the *DsRed* cassette for subsequent removal of the cassette. We reared all flies at 25°C, 60–75% relative humidity, and a 12-hour light-dark cycle on standard cornmeal–molasses–agar medium. Before experimentation, we reared the flies for 2 generations at controlled densities (5 males and 5 females per vial allowed to lay eggs for 2 days). We used 3–5-day-old flies for all experiments.

### Quantification of organismal phenotypes

#### Starvation stress resistance

We used Drosophila activity monitors to measure starvation stress resistance. We placed one fly per tube containing starvation medium (1.5% agar in distilled water) and ran the assay for 4 days in accordance with previous work (Chiu *et al*., 2010), with a total of 64 flies per sex per genotype. We obtained activity bout data using Shiny-R DAM (Cichewicz and Hirsch, 2018) and used the time of last activity bout as the time of death.

#### Response to heat shock

The day before measuring the response to heat shock, flies of each genotype were lightly anesthetized with CO_2_ and sorted into single-sex groups of 20 individuals in standard vials containing 5 ml food. On the day of the heat stress exposure, flies from each replicate vial were transferred without anesthesia into vials without food and placed in an incubator at 37°C (±0.5°C) for 180 minutes. After heat stress exposure, flies were immediately transferred to vials containing 5 ml of standard cornmeal–agar–molasses medium and returned to the 25°C incubator for 24 hours. The percentage of surviving flies per vial was recorded 24 hours after the 3 hours heat shock. A fly was considered alive if it could move when the vial was gently tapped. We performed 5 replicates per genotype and sex.

#### Activity and sleep

We assessed total activity and proportion of sleep during the day and night (Shaw *et al*., 2000; Hendricks *et al*., 2000) using Drosophila Activity Monitors (TriKinetics). We ran the assay in accordance with previously published work (Chiu *et al*., 2010) and recorded data for 5 days on at least 64 flies per sex per genotype. We processed and analyzed the data using Shiny-R DAM (Cichewicz and Hirsh, 2018).

### Statistical analyses of organismal quantitative traits

We assessed mean differences of phenotypic values using factorial, fixed effects ANOVA models: *Y* = *μ* + *Genotype* + *Sex* + *Genotype* × *Sex* + *ɛ*, where *Y* is the phenotype, *μ* is the overall mean, and *ɛ* is the residual (error) variance. All analyses were performed using SAS Studio release 3.71 (SAS Institute, Cary, NC, USA).

### RNA sequencing

To prepare libraries for RNA sequencing, we collected 2 replicates/sex/genotype of 50 flies each, between 1 and 3 PM, and flash froze them on dry ice in 15 ml Falcon tubes (Thermo Fisher Scientific, Waltham, MA, USA). The flies were decapitated using a strainer (Carolina Biological Supply Company, Burlington, NC, USA) for head collections. The heads were collected on a dry ice-cooled fly pad and placed in 2 ml pre-filled tough microfuge tubes with glass beads. Total RNA was extracted using the Direct-Zol microprep kit RNA extraction protocol (Zymo Research, Irvine, CA, USA). The heads were homogenized in a bead mill (Thermofisher) for 1 minute at 4 m/s, after which the RNA was eluted with 15 μl water. We depleted ribosomal RNA using the NuQuant + UDI, Drosophila AnyDeplete kit (Tecan, Männedorf, Switzerland) and prepared bar-coded cDNA libraries for paired end sequencing on an S1 flow cell on the NovaSeq 6000 platform (Illumina, San Diego, CA, USA) using a 2 × 150 bp chemistry with an average of 36 million raw reads resulting in 20 million aligned reads and an average read length of 250–300 bp, as described previously (Johnstun *et al*., 2021).

### Analysis of RNA sequences

We performed the initial steps of raw read processing and normalization of expression as previously described (Johnstun *et al*., 2021). Briefly, we used the AfterQC pipeline (Chen *et al*., 2017) to trim adapters, detect abnormal polynucleotide sequences, filter for low quality (Q < 20) and short (<35 nt) sequence reads, and generate basic sequence quality metrics. We used the bbduk command from the BBTools package (Bushnell, 2018) to detect rRNA contamination. We aligned high-quality sequence reads to the *D. melanogaster* reference genome release 6 (version 6.13) using GSNAP aligner (Wu and Nacu, 2010) and mapped unique alignments to genes using the Subread package (Liao *et al*., 2013). We used GeTMM (Smid *et al*., 2018) to normalize filtered expression counts. Differential expression between null and CSB within each sex was assessed using separate contrast statements after fitting a GLM model to the normalized counts in edgeR (Robinson *et al*., 2010). Prior to fitting, genes were filtered for expression using default parameters part of the filterbyexp function. Raw *P*-values derived from GLM likelihood ratio Test were adjusted for multiple hypothesis testing using Benjamini–Hochberg false discovery rate (BH-FDR) method and genes with BH–FDR < 0.05 were considered statistically significantly differentially expressed. We performed functional enrichment analysis on the differentially expressed genes for biological processes and Reactome pathways using Panther (Mi *et al*., 2017).

## Results

### Effects of Obp56h alleles on organismal phenotypes

We constructed an *Obp56h* deletion line using CRISPR/*Cas9* technology (Fig. 1a) and confirmed the deletion of *Obp56h* with appropriate primers for Sanger sequencing (Supplementary Table 1) and the absence of its transcript from whole genome RNA sequences (Fig. 1b). Removal of *Obp56h* did not affect sex ratio or viability.

**Fig. 2.**
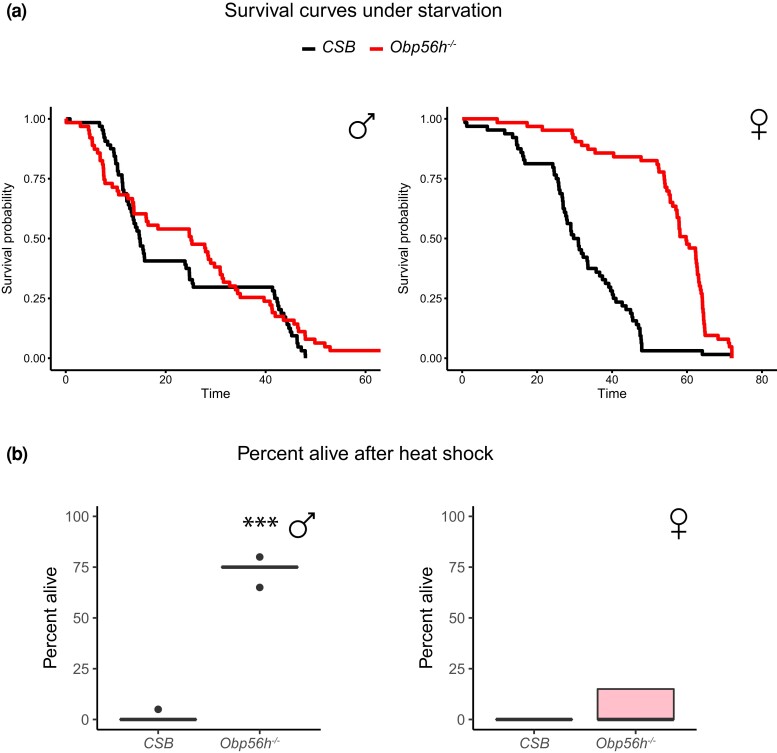
Pleiotropic effects of *Obp56h* alleles on fitness-related quantitative traits. a) Survival curves under starvation stress. The black survival curve represents the *CSB* control, and the red survival curve represents the *Obp56h*^−/−^ flies. b) Heat shock survival. Males are indicated in blue and females in pink. ***P < 0.0001 (Supplementary Table 2).

We assessed the effects of *Obp56h* deletion on starvation stress resistance, heat stress resistance, locomotor activity, and sleep traits (Supplementary Table 2). *CSB* females were more resistant to starvation stress than males. Deletion of *Obp56h* did not affect starvation resistance of males but greatly increased starvation stress resistance of *Obp56h*^−/−^ females doubling their mean survival time (*P* < 0.0001; Fig. 2a). *Obp56h*^−/−^ females were as sensitive to heat stress as the *CSB* control with few survivors. Males, however, were resistant to heat shock with about 75% survival compared to *CSB* [(*P* < 0.0001); Fig. 2b]. Thus, deletion of *Obp56h* has sexually dimorphic effects on stress resistance, with protective effects for females under starvation stress and for males under heat shock conditions.

**Fig. 3.**
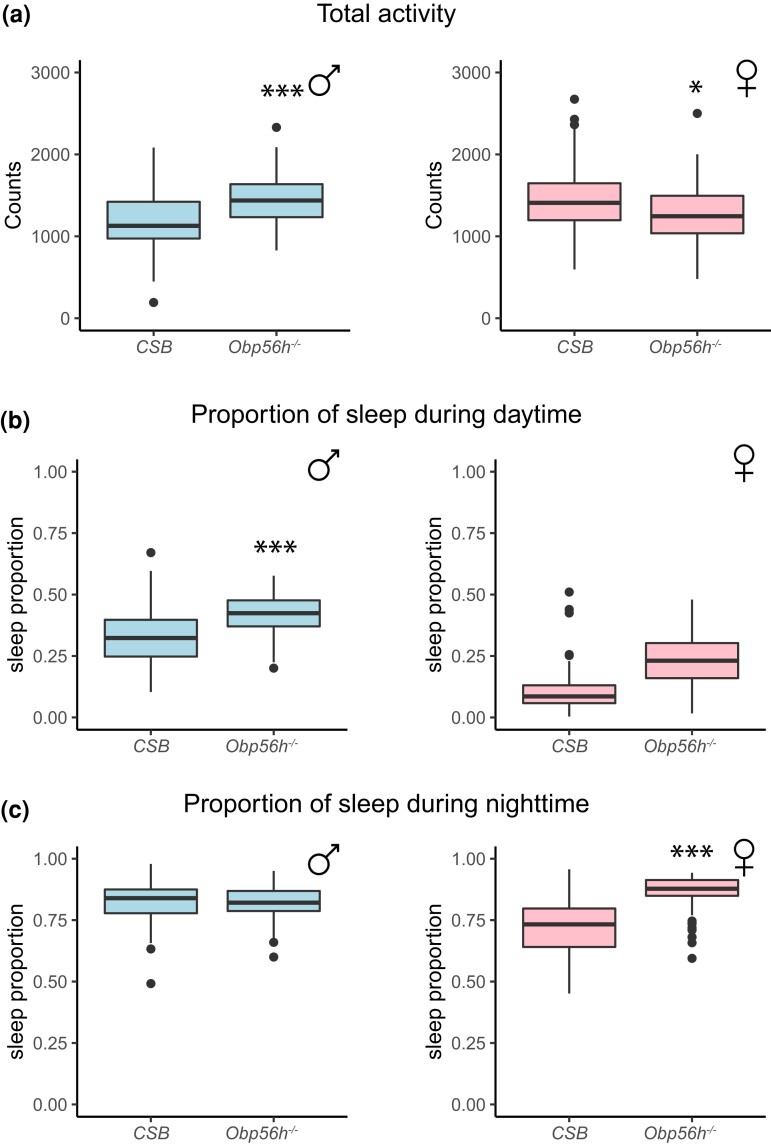
Effects of *Obp56h* alleles on activity and sleep phenotypes. Box plots showing total activity (a), sleep proportion during the day (b), and sleep proportion during the night (c) for sexes separately. *P < 0.05; **P < 0.001; ***P < 0.0001 (Supplementary Table 2).

Deletion of *Obp56h* also affects locomotion and sleep. Males show an increase in total locomotion, and this increased activity is accompanied by an extended sleep proportion during the day (Fig. 3a; Supplementary Table 2). In contrast, females showed reduced locomotor activity and increased sleep proportions during the day and night (Fig. 3b and c; Supplementary Table 2).

### Effects of Obp56h alleles on genome-wide gene expression

**Fig. 4.**
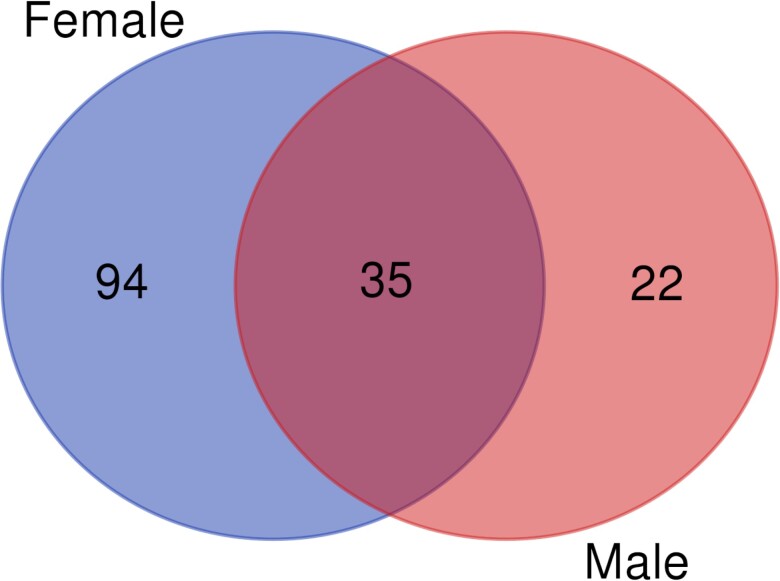
Venn diagram of differentially expressed genes across both sexes in *Obp56h*^−/−^ flies compared to the control.

To gain insights into the cellular processes that might underlie the observed sexually dimorphic pleiotropic effects of *Obp56h*, we identified differentially expressed genes in the *Obp56h*^−/−^ flies compared to the *CSB* control. The *D. melanogaster* transcriptome is highly modular (Ayroles *et al*., 2009). Mutations that disrupt a gene associated with complex organismal phenotypes generate *trans*-regulatory effects resulting in changes in co-regulated ensembles of genes (Anholt *et al*., 2003; Arya *et al*., 2010; Boyle *et al*., 2017; Anholt and Mackay, 2018). We obtained whole transcriptome profiles on heads of males and females separately (Supplementary Table 3). At a false discovery rate (FDR) < 0.05, we identified 130 differentially expressed genes in females and 57 in males (Supplementary Table 4; Fig. 4). Thirty-five genes were common among both sexes, including *CG17224*, predicted to encode a uridine phosphorylase (Gaudet *et al*., 2011), *DNAJ-H*, which encodes a chaperone heat shock protein (Gaudet *et al*., 2011; Whitaker *et al*., 2013), genes associated with antimicrobial immune responses, including *def, Attb, DptA, CecA1* and *pirk,* and predicted genes with unknown functions. Long noncoding RNAs, snoRNAs, and tRNAs also show differential expression in both males and females. Most of the male-specific genes are genes of unknown function. As expected, expression of *Obp56h* was absent in both *Obp56h*^−/−^ males and females compared to the *CSB* control.

Along with lncRNAs and snoRNAs, several *snRNA*s are differentially expressed in females, which are associated with control of mRNA splicing (Wilkinson *et al*., 2020). Genes that encode cuticular proteins (*Cpr49Ae*, *Cpr100A, Acp1*, and *Edg91*) are differentially expressed in females, along with *CG12895*, which is associated with the assembly of succinate dehydrogenase, ribosomal proteins (*RpS2*, *RpS3*, *RpS15Ab*, *RpL3*, *RpL7* and *RpL28*), and components of the mitochondrial electron transfer chain (*CG40472*, *Mt:ND2* and *Mt:ND6*) (Supplementary Table 4).

Several transcripts that are responsive to stress are also differentially expressed. In females, these include *TotA*, *TotC*, and *TotM*, and in males *TotX*, members of the Turandot family of peptides that are secreted in response to stress (Ekengren and Hultmark, 2001; Brun *et al*., 2006), in addition to the heat stress-related *DNAJ-H* chaperone (Supplementary Table 4). *Obp56h*^−/−^ females show differential expression of *Lst*, which encodes the peptide hormone limostatin that is released by the corpora allata during starvation and suppresses insulin secretion (Alfa *et al*., 2016). *CCHa2*, which encodes a ligand for the CCHamide-2 receptor that stimulates food intake (Ida *et al*., 2012; Ren *et al*., 2015), is also differentially expressed in *Obp56h*^−/−^ females (Supplementary Table 4).

## Discussion

We used CRISPR/*Cas9*-mediated gene deletion to assess pleiotropic effects on fitness-related quantitative traits and changes in genome-wide gene expression upon deletion of *Obp56h*. We chose *Obp56h* for its favorable properties for CRISPR/*Cas9* gene editing and because previous studies suggested that this gene might have pleiotropic effects on the transcriptome and organismal phenotypes (Swarup et al., 2011, 2014; Shorter et al., 2016). We found considerable sexual dimorphism in the effects of *Obp56h* deletion on survival under starvation and heat stress, locomotor activity, and day and night sleep. *Obp56h*^−/−^ mutants show a strong sexually dimorphic effect on starvation stress survival, with females being more resistant to starvation stress than the control. In contrast, *Obp56h*^−/−^ females, but not males, are highly sensitive to heat stress. The molecular mechanisms and cellular processes that give rise to these sexually dimorphic effects that result from deletion of *Obp56h* remain to be determined. We cannot exclude additional organismal phenotypes that may be affected by deletion of *Obp56h*. Whole-genome transcript analyses showed common genes affected by *Obp56h* deletion in both sexes as well as sexually dimorphic differences in differential gene expression (Supplementary Table 4). The mechanisms by which mutations in a focal gene cause correlated changes in coregulated genes are not known. Such changes may change the function of neurons or glia in the brain and could possibly also result in trans-cellular effects on gene expression.

It is not surprising that there are differences in differential gene expression in *Obp56h* knock-out flies between males and females. There is a vast body of evidence in the literature that the genetic architectures that underlie complex traits are distinct between males and females. What gives rise to these differences is a big question in quantitative genetics that has not yet been resolved.

Differential expression of *Antennal dehydrogenase* (*Antdh*) in both sexes and *Obp56a* and *Obp19d* in females (Supplementary Table 4) is consistent with a previously reported role for *Obp56h* in chemosensation (Swarup *et al*., 2011, 2014). A previous study, in which expression of *Obp56h* was reduced through RNA interference, also found up-regulation of antimicrobial response peptides and Turandot peptides in heads of males and females (Shorter *et al*., 2016). This study also reported changes in cuticular pheromones in whole flies upon RNAi-targeted inhibition of *Obp56h* under a *Dll-GAL4* driver (Shorter *et al*., 2016).

It is intriguing to note that the increased starvation stress resistance of *Obp56h*^−/−^ females is consistent with a reduction in transcript abundance for limostatin, which suppresses insulin production, thereby allowing increased levels of insulin during starvation stress (Alfa *et al*., 2016), while up-regulation of the orexigenic peptide CCHa2 enhances appetite (Ren *et al*., 2015). We can speculate that increased mitochondrial metabolism, as reflected by up-regulation of electron transfer components, might offer protection during starvation stress by promoting energy generation to maintain cellular integrity. It is possible that changes in gene expression that promote resistance to starvation stress are not protective against heat stress.

The ligand(s) for *Obp56h* in the brain is unknown but could be hydrophobic metabolites, which play a role in fundamental cellular processes. In addition, the link between *Obp56h* and gene regulation remains unknown. Altered expression of lncRNAs that may control gene expression, snRNAs that regulate splicing (Wilkinson *et al*., 2020), and snoRNAs associated with ribosomal function (Reichow *et al*., 2007) may be part of the mechanisms that regulate gene expression upon deletion of *Obp56h*.

The pleiotropic fitness effects of this member of the *Obp* family are likely not unique to *Obp56h* but may also pertain to other members of this rapidly evolving gene family. The results from this study, together with previous observations by us (Arya *et al*., 2010; Johnstun *et al*., 2021) and others (McGraw *et al*., 2004; Findlay *et al*., 2008; Sun, Larter, *et al.*, 2018), contribute to the mounting evidence that the functions of OBPs extend well beyond chemosensation and affect a variety of fundamental cellular processes.

## Supplementary Material

jkac307_Supplementary_Data

## Data Availability

RNA sequence data have been deposited in the GEO repository under accession number GSE215148. All raw data and code are available at: https://github.com/snehamokashi/Obp56h_KO_vs_CSB.git. Supplemental material available at *G3* online.

## Notes

*Communicating editor: S. Lott*
