# Supplementary material for: Pleiotropic fitness effects of a Drosophila odorant-binding protein
Source: G3 (Bethesda). 2022 Dec 1;13(2):jkac307. doi: 10.1093/g3journal/jkac307 (PMC9911060; doi:10.1093/g3journal/jkac307)
Supplement: jkac307_Supplementary_Data [file jkac307_supplementary_data.zip › Supplementary_Table_Legends_G3-2022-403887.docx]

**Supplementary Information**

**Supplementary Table 1. Primers used for generation and validation of the *Obp56h* null allele.**

**Supplementary Table 2. Effects of *Obp56h* null allele on organismal quantitative traits.** Genotype means and significance of differences from *CSB* (*t*-tests).

**Supplementary Table 3. RNA sequencing raw data. *CSB*: Canton S B control.** Obp56h_KO: Obp56h null allele M denotes males and F denotes females, and 1 and 2 indicate replicates 1 and 2, respectively. (A) Numbers of reads per gene. (B) Filtered and normalized counts/million reads.

**Supplementary Table 4. RNA sequencing differential expression analysis.** (A) Genes differentially expressed when *Obp56h* is knocked out in females. (B) Enrichment analysis by Gene Ontology analysis for the female dataset. (C) Gene differentially expressed when *Obp56h* is knocked out in males. (D) Enrichment analysis by Gene Ontology analysis for the male dataset. (E) Genes differentially expressed in both males and females.
